# Pulsed THz radiation under ultrafast optical discharge of vacuum photodiode

**DOI:** 10.1007/s12200-024-00123-5

**Published:** 2024-06-13

**Authors:** Aleksandr Ushakov, Kseniia Mamaeva, Leonid Seleznev, Georgy Rizaev, Vladimir Bukin, Timophey Dolmatov, Pavel Chizhov, Vladimir Bagdasarov, Sergey Garnov

**Affiliations:** 1 Prokhorov General Physics Institute of the Russian Academy of Sciences, Moscow, 119991 Russia; 2 Physical Institute, Russian Academy of Sciences, Moscow, 119991 Russia; 3 Moscow Institute of Physics and Technology, Dolgoprudny, 141700 Russia; 4 Russian Institute for Scientific and Technical Information, Moscow, 125190 Russia

**Keywords:** Electron emission, Vacuum photodiode, Ultrafast excitation, Terahertz

## Abstract

In this paper, we first present an experimental demonstration of terahertz radiation pulse generation with energy up to 5 pJ under the electron emission during ultrafast optical discharge of a vacuum photodiode. We use a femtosecond optical excitation of metallic copper photocathode for the generation of ultrashort electron bunch and up to 45 kV/cm external electric field for the photo-emitted electron acceleration. Measurements of terahertz pulses energy as a function of emitted charge density, incidence angle of optical radiation and applied electric field have been provided. Spectral and polarization characteristics of generated terahertz pulses have also been studied. The proposed semi-analytical model and simulations in COMSOL Multiphysics prove the experimental data and allow for the optimization of experimental conditions aimed at flexible control of radiation parameters.

**Graphical Abstract:**

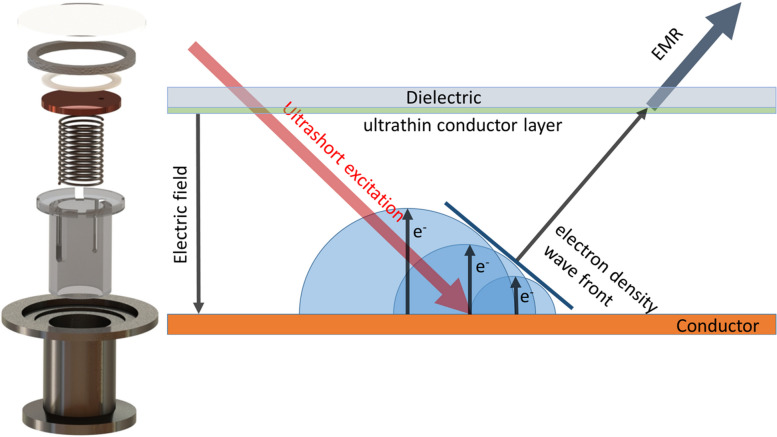

## Introduction

Generation and registration of terahertz (THz) electromagnetic radiation (EMR) is one of the key scientific direction during last three decades [1] due to its different applications in science and techniques. Pulsed THz radiation sources are of particular interest in time-domain spectroscopy applications [2] and time-of-flight imaging [3–5]. Sources of THz radiation based on the laser radiation interaction with matter are widespread. Such sources generally include laser exited photoconductive antennas [6, 7], laser-induced plasma [8–14], laser illuminated nonlinear crystals [15–17] and others. However, development of new types of THz sources, including those with tunable generated spectrum, can open up new horizons in basic and applied research.

One of the possible methods for obtaining an EMR is based on oblique illumination of the flat surface by X-ray radiation [18]. A further increase of the EMR power is possible via accelerating of emitted electrons by external electric field in vacuum photodiode [19, 20]. The generation of THz radiation during oblique incidence of laser radiation on a semiconductor was previously demonstrated due to the photo-Dember effect [21] and during irradiation of a diamond-based photoconductive antenna with longitudinally applied electric field [22]. However, a generation method by using of vacuum photodiode is primarily aimed at generating ultra-wideband waves in the microwave range, which explains the great interest in such sources from the point of view of radar and power effects on electronics [23]. The low cost of generating a static electric field power supplies for amplification of output EMR power compared to expensive laser systems make these sources extremely promising for generating ultrawideband electromagnetic pulses. By changing the parameters of the emitting element (diode), choosing different light sources and different power sources for the diode, it is possible to obtain a wide range of devices that generate an electromagnetic pulse in the microwave range [24]. In the case of using an anode opaque to electrons, it becomes possible to shorten the tail of the EMR pulse [25]. It should be noted that one of the factors determining the dynamics of EMR generation is the duration of the laser pulse initiating electron emission. The use of ultrashort laser radiation makes it possible to receive EMR pulses with spectral components in the THz region.

In this paper, we first present an experimental demonstration of THz radiation pulse generation with energy up to 5 pJ under the ultrafast optical discharge of a vacuum photodiode. We use femtosecond optical excitation of metallic copper photocathode in flat vacuum photodiode for the generation of ultrashort electron bunch exposed to external electric field up to 45 kV/cm for the photo-emitted electron acceleration. In Section 2, semi-analytical modeling and numerical simulations in COMSOL Multiphysics, describing the process of EMR generation are suggested. In Section 3, construction of vacuum photodiode and details of experimental setup are provided. In Section 4, experimental results and a comparative study with modelling results are presented. Terahertz pulse energy vs. emitted charge density, incidence angle of optical radiation, optical beam diameter and applied electric field dependencies are demonstrated. A comparative study of spectral characteristics received by using of different experimental techniques and simulations is also provided. Besides, the polarization state of generated electromagnetic pulses has been experimentally determined. Finally, we conclude the results and the perspective of photo emissive sources for generation of THz radiation in Section 5.

## Model and numerical simulation

### Model of EMR generation

**Fig. 1:**
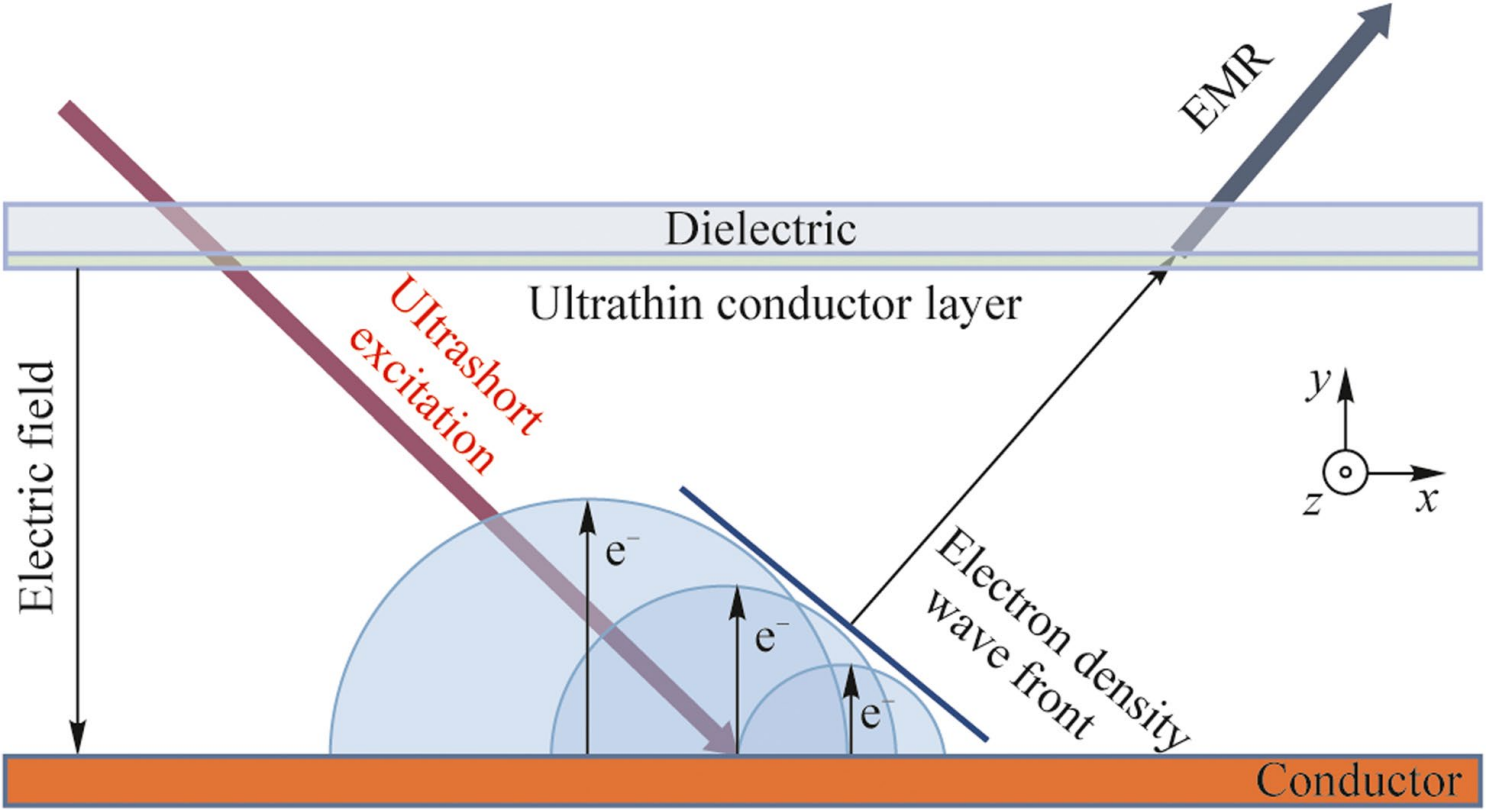
Scheme of EMR radiation generation under electron emission in vacuum photodiode

For the understanding of EMR generation process a flat photodiode with an optical excitation falling at the incident angle *θ* to the cathode surface normal is considered (see Fig. 1). Thereafter emitted electrons are accelerated under an external electric field and directed to the anode. The front of electron emission wave spreads across the cathode surface with the velocity *v* = *c*/sin*θ*, thus the generated EMR has a directivity at the angle corresponding to the reflection of the incident optical excitation. The anode is a flat dielectric material transmissive for optical and generated EMR but opaque for emitted electrons.

According to Ref. [18], the magnetic field strength *H* is determined by time derivative of the dipole momentum *P* and the tangent of the incidence angle *θ* by

1 $$H=\dot{P}\text{ tan}\theta .$$

We assume for relatively low energies (nonrelativistic regime) of electrons during emission under ultrashort optical excitation from cathode and following acceleration in the cathode–anode gap that the dipole momentum derivative may be found as a function of a set current density. This current is determined by Coulomb repulsion only and the interaction of electrons with the laser and the generated field is negligible. In this case the magnetic field strength may be described by

2 $$H=\text{tan}\theta \times \left\{\begin{array}{cc}\frac{{\epsilon }_{0 }e {U}^{2}}{m {d}^{2}}\left(\xi -\frac{{\xi }^{2}}{2}\right)t,& 0\le t<\tau ,\\ \frac{{\epsilon }_{0}e{U}^{2}}{m {d}^{2}}\left(\xi -\frac{{\xi }^{2}}{2}-\frac{1}{2}\right)t+\frac{2{\epsilon }_{0}m{d}^{2} }{e{t}^{3} },& \tau \le t<{t}_{a},\\ 0,& {t}_{a}\le t,\end{array}\right.$$

where *e*, *m* –the charge and the mass of an electron, *U* – the applied voltage, *d* – the gap distance between the cathode and the anode, *ϵ*_0_ – the electric constant, *ξ –* the ratio of emitted charge density *σ* to the one stored on the cathode, and *τ* and *t*_*a*_ – times of flight from the cathode to the anode (in assumption of a zero electron start velocity) of the first and the last of the emitted electrons correspondingly. Values *ξ, τ*, and *t*_*a*_ may be found according to the following formulas:

3 $$\begin{array}{l}\xi =\frac{\sigma d}{{\epsilon }_{0}U},\\ \tau =\sqrt{\frac{2m{d}^{2}}{eU}},\\ {t}_{a}=\sqrt{\frac{2m{d}^{2}}{eU(1-\xi )}}.\end{array}$$

**Fig. 2:**
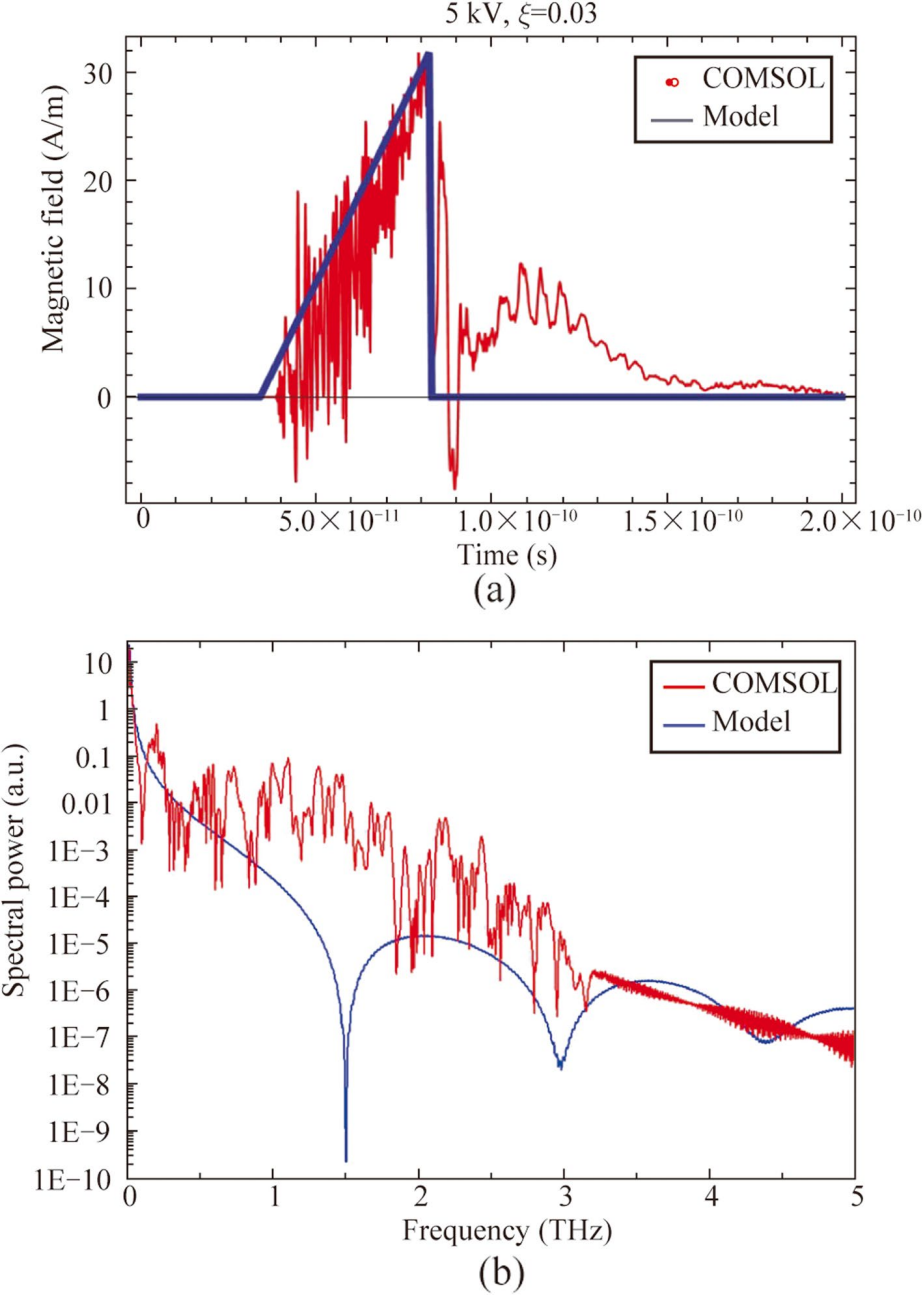
**a** Waveforms of magnetic field corresponded to calculations based on Eq. (2) (blue line) and numerical simulations in COMSOL Multiphysics (red line) and **b** corresponded Fourier spectra

This simple model describes the waveform generated during electron emission in the vacuum photodiode. Timescale of *τ* has a typical value 10–100 ps, at the same time, shorter time interval of decelerating of the electron bunch on anode (*t*_*a*_ – *τ*) leads to < 10 ps values. Mentioned condition is necessary for THz radiation generation under electron emission in vacuum photodiode. In semi-analytical model the initial electron beam duration is significantly small. In general, a typical timescale of generation process is in the order of 10 − 100 picoseconds, as it is presented in Fig. 2. Thus, excitation laser pulse durations equal to 150 fs is significantly less in comparison with photoelectron dynamics in overall generation process and may be neglected. Maximum generated spectral component in model is determined by re-radiation of electron bunch during stopping on the anode. At this moment duration of electron bunch is mainly governed by Coulomb repulsion. In general, laser pulse duration could significantly influence the electron bunch generation and corresponding THz spectrum. Rising in laser pulse duration up to comparable with electron bunch dynamics timescale should decrease the maximum spectral component. First publications on using photo emissive sources demonstrated microwave radiation, due to laser pulse durations were pico or nanoseconds. In our experiments laser pulse duration was fixed at bandwidth-limited level –150 fs, due to the photoelectron production efficiency of copper cathode is weak and nonlinear, thus significantly dependent on laser beam intensity (duration). We have tried to increase the optical excitation pulse duration, however, it drastically decreased the THz yield from photodiode and did not provide any information about THz spectra change.

### Simulation in COMSOL Multiphysics

We performed simulations in COMSOL Multiphysics to compare with experimental data and confirm our assumptions about the above analytical model. Initial and boundary conditions have been assumed similar to the presented in Fig. 1. In COMSOL simulations initial electron bunch duration is set analogically to analytical model. Then the electron emission only in the region between the cathode and the anode has been specified. In simulations 2D + time distributions of the magnetic field have been obtained. Thereafter calculated EMR waveforms in a point at the distance of 2 cm from the output quartz anode are used for a comparison with the analytical model. One of magnetic field waveforms is presented (see Fig. 2).

This distribution corresponds to the *z*-component of the magnetic field *H* due to other components are equal to zero. In Fig. 2, we also introduce a magnetic field waveform based on Eq. (2) corresponding to the analogical parameters (*U* = 5 kV, *d* = 1 mm, *ξ* = 0.03). Corresponded Fourier spectra are also presented and they demonstrate nonzero output in THz region. One can see that these two waveforms are in good agreement, some differences are connected with refraction of EMR from the quartz anode and the cathode, which is not included in the analytical model calculations (Eqs. (1) − (3)).

## Experimental detail

### Vacuum photodiode

**Fig. 3:**
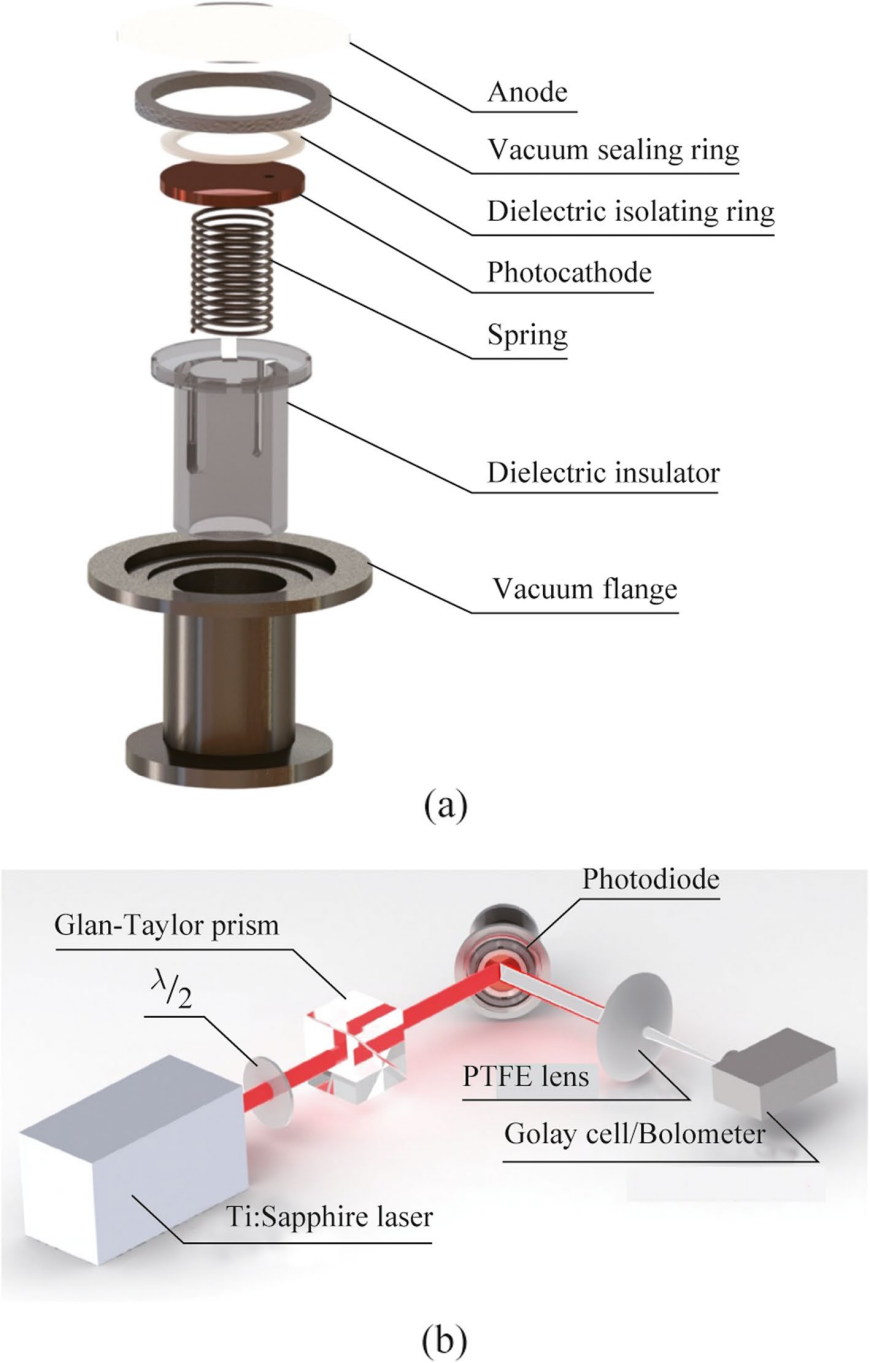
**a** Construction of vacuum photodiode; **b** scheme of experimental setup

The exploded view of the photodiode designed to experimentally study the process of EMR generation under electron emission is shown in Fig. 3a. The developed photodiode consists of several parts. An anode – a crystalline quartz with indium tin oxide (ITO) film, coated onto vacuum side. A vacuum elastomeric ring serves for sealing the vacuum photodiode; a dielectric ring plays a role of a spacer (polytetrafluoroethylene spacers 1 mm thick) to maintain the gap between the anode and a photocathode. The photocathode is a copper disk with a small hole (~ 1 mm in a diameter) for pumping of the gap between anode and cathode. A metallic spring is set to ensure electrical contact between the photocathode and the vacuum flange. This spring performed the role of a clamping mechanism to maintain a constant gap between the photocathode and the anode. A dielectric insert is mounted for a positioning the photodiode during assembly. Whole photodiode components are collected in a vacuum flange—an adapter KF40 − KF25, which was directly connected to the pumping system. An atmospheric air press onto the anode outside thus sealing of a photodiode is realized. A vacuum pump system maintains a pressure inside the photodiode lower than 10^−6^ mbar.

**Fig. 4:**
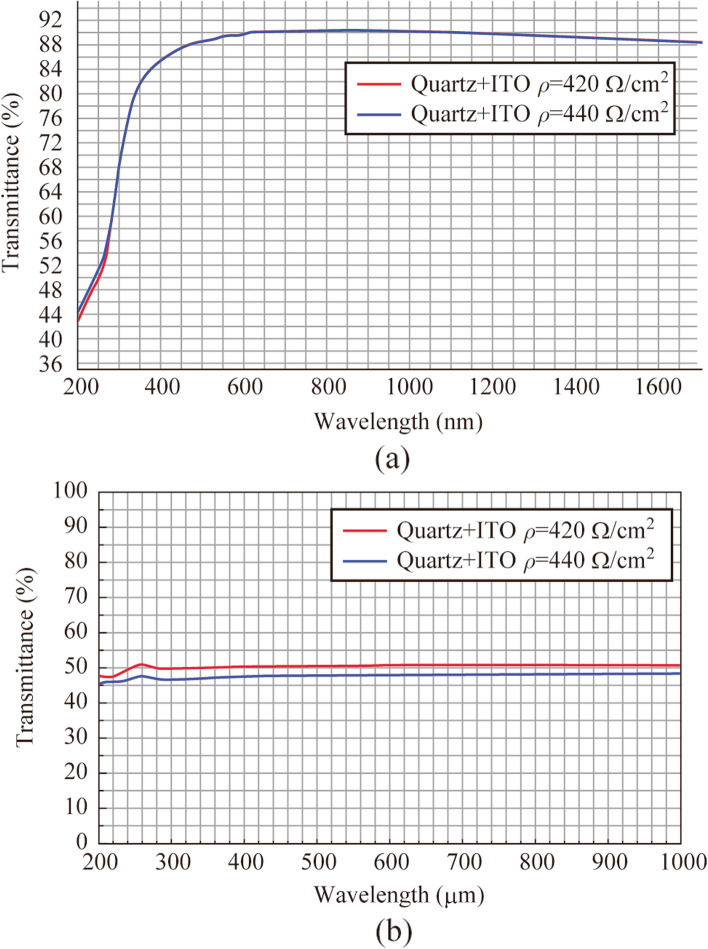
Transmission spectra of Quartz anode with ITO film in **a** optical and **b** THz region

An external electric field is applied between the cathode and the anode for the acceleration of electrons emitted from the cathode under action of optical excitation (see Fig. 3b). A positive voltage is applied at ITO film and negative voltage is applied at vacuum flange connected with photocathode. Anode properties are chosen for the possibility of effective generation of electron emission on the one hand and for the transmission of THz waves outside the photodiode on the other hand. To this end the thickness of ITO film is chosen to be around 100 nm with corresponding surface electric resistance of 420−440 Ω/cm^2^. The anode transmission spectra for optical (see Fig. 4a) and THz (see Fig. 4b) radiation are provided below. The anode on an ITO-coated crystalline quartz substrate has a transmittance of 90% at a wavelength of 800 nm, and also is about 50% in the THz range.

### Experimental setup

The generation and characterization of EMR pulses during photoelectron discharge of a vacuum diode was achieved using a setup (see Fig. 3b). The initiating source for this process was the radiation from a Ti:Sapphire laser system Coherent Legend Elite Pro (central wavelength 800 nm, 150 fs pulse duration, up to 2.7 mJ pulse energy, a beam diameter of 12 mm at level 1/e^2^). The pulse repetition rate was tunable in the range of 4 to 1000 Hz. To regulate the energy and control the laser radiation polarization state a Glan–Taylor prism and a half-wave plate were inserted into the beam. Thereafter the radiation was directed toward the input window of the flat photodiode at an angle *θ* to the normal. After passing through the anode, the laser radiation was partially absorbed by the cathode, which induced the emission of photoelectrons from its surface. These electrons were then accelerated toward the anode by an external constant electric field (in experiments it was varied in region 0–45 kV/cm), and upon reaching the anode, they were decelerated in the near-surface layer, resulting in the emission of secondary radiation. The output terahertz radiation was collected and focused with a polytetrafluoroethylene (PTFE) lens into a detector. For the control of the polarization state of the THz radiation, a wire-grid polarizer was set in the beam path in front of the detector. We have also performed monitoring of the emitted electric charge by connection of an oscilloscope in the electric circuit to the photodiode.

The detector was a receiving system of electromagnetic radiation (in following named as bolometer), based on a closed cycle cryo-refrigerator model RS-CCR-1-12T-1 + 0.3-3T-0.1 (manufactured by Skontel LLC). The operating spectral range of the main detector channel was 0.3 − 15 THz. At the same time, a filter made of high-density polyethylene was placed on the input window of the detector, which did not transmit optical radiation. In several experiments when THz energy exceeded the saturation threshold of the bolometer (about 10^−13^ J) a set of broadband attenuators (manufactured by Tydex LLC) with uniform transmission in the range 40 ÷  > 1000 μm was located in front of the input window of the bolometer. We also used a Golay cell (GC-1P, manufactured by Tydex LLC) instead of the bolometer in several experiments, when the registered energies of the THz radiation were above its minimal detection threshold (~ 2 × 10^−13^ J).

## Results

First, we have performed a verification that the generated THz radiation has corresponded to the ultrafast optical discharge of the vacuum photodiode. The incidence angle of the optical radiation and the corresponding expected angle of THz emission was set at 45°. The bolometer with the set of attenuators and the PTFE lens (*f* = 10 cm) was mounted on the axis corresponding to the expected THz generation direction. This direction has also been proven by registration of the maximal THz signal. This signal has been observed only when applying external DC voltage to the photodiode and the action of optical excitation on the cathode simultaneously. The application of external constant electric field only or the action of laser radiation on the cathode only did not lead to the detection of any significant THz signal.

We have estimated the angular distribution of THz radiation from vacuum photodiode. The main part of the radiation (> 80%) spreads in azimuthal angle ± 20° from the optical axis collinear to the reflection angle of optical excitation. However, due to low energy level of the THz pulses we have to use a PTFE lens for collection of the THz radiation, thus direct measurements of THz angular distribution were complicated. In general, generated THz pulses should be collimated, however, bend of anode and roughness of cathode can lead to additional divergence except of standard diffraction.

In experiments using a bolometer as a detector we have revealed that the energy of recorded pulses does not depend on the pulse repetition frequency in the range 10 − 1000 Hz. Further measurements with bolometer have been done at 100 Hz repetition rate. Experiments with Golay cell have been done at 1 kHz laser repetition rate with gated operation on 15 Hz modulation frequency.

**Fig. 5:**
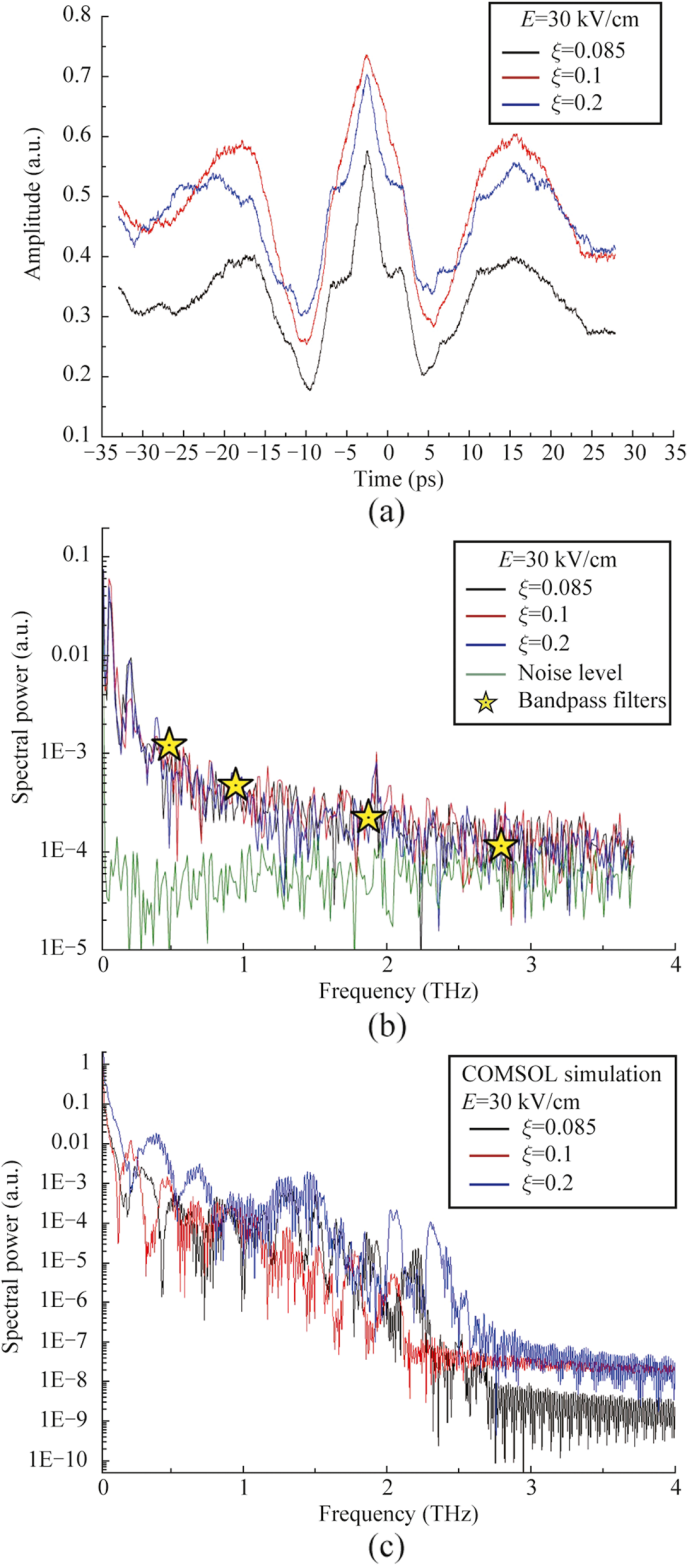
**a** Correlation functions of THz pulses and **b** corresponded experimental and **c** simulated in COMSOL Multiphysics Fourier spectra. Yellow stars in **b** corresponds to experimentally obtained data by using of bandpass THz filters

We have performed measurements of THz signal autocorrelation function (see Fig. 5) by using of a Michelson interferometer with a movable mirror in one of the arms and a high resistive silicon beamsplitter, as it was presented in Ref. [26]. In our work we use two types of incoherent detectors: Golay cell and bolometer. Physical mechanisms of these detectors do not provide any information about field amplitude, phase and spectra. Application of coherent detection technique, for example electro-optical sampling (EOS) or air-biased coherent detection (ABCD) provides an information about amplitude and phase of THz field. Using of fast Fourier transform, it also provides information about spectrum. There are several manuscripts devoted to generation of ultrabroadband THz radiation from laser induced plasma, which demonstrate comparative analysis of THz spectral measurements by using of EOS, ABCD and FTIR-technique (bolometer with interferometer Michelson as an autocorrelator) for example in Ref. [26]. These measurements demonstrate that autocorrelation technique is applicable for detection of ultrabroadband THz pulse spectrum and provides wider spectral detection in comparison with the others. This allows to use this detection technique in our experiments due to THz pulses are generated only in the moment of the vacuum photodiode discharge by laser pulses, this process is repeatable and similar to generation of THz pulses in laser induced plasma or photoconductive antenna. Besides, periodic oscillations, symmetry of correlation functions and reproducibility of experimental data characteristic for pulsed THz detection with FTIR-technique have also been observed in experiments. Thus, in the term of coherence of electromagnetic pulses from photodiode a coherent detection of this radiation should be possible. Since the energy of THz pulses is in the order of susceptibility of EOS detector or less, we use autocorrelator in our setup. Since the parameter *ξ* and the applied voltage are related to each other in terms of the temporal dynamics of the flight of electron beams and the emission spectrum, we decided to focus on measuring the correlation functions for different values of the parameter *ξ*. Measured autocorrelation functions have demonstrated a low variation of pulse duration and shape as a function of *ξ*. The corresponding THz emission spectra also show minor changes in shape and spectral composition. Simulated in COMSOL Multiphysics Fourier spectra also show a decreasing dependence of spectral power with increasing frequency and a similar upper cutoff at approximately 2.5 − 3 THz frequency. We have also check observed signal by using a set of bandpass THz filters (maximum spectral transmission at frequencies 0.5, 1, 2, and 3 THz with bandwidth 0.1 THz, manufactured by Tydex LLC). For all these filters detected signal was not equal to zero, with maximum signal observed using the 0.5 THz bandpass filter (see Fig. 5b). The maximum of generated spectra is located in low frequency region both for experimental and numerically simulated spectra. Direct comparative analysis of experimental and numerically simulated spectra demonstrates monotonically decrease behavior of spectrum with increasing frequency. Some differences may be connected with aperture diffraction effect, chromatic aberration in PTFE lens and nonuniform response of bolometer which are not considered in simulations.

**Fig. 6:**
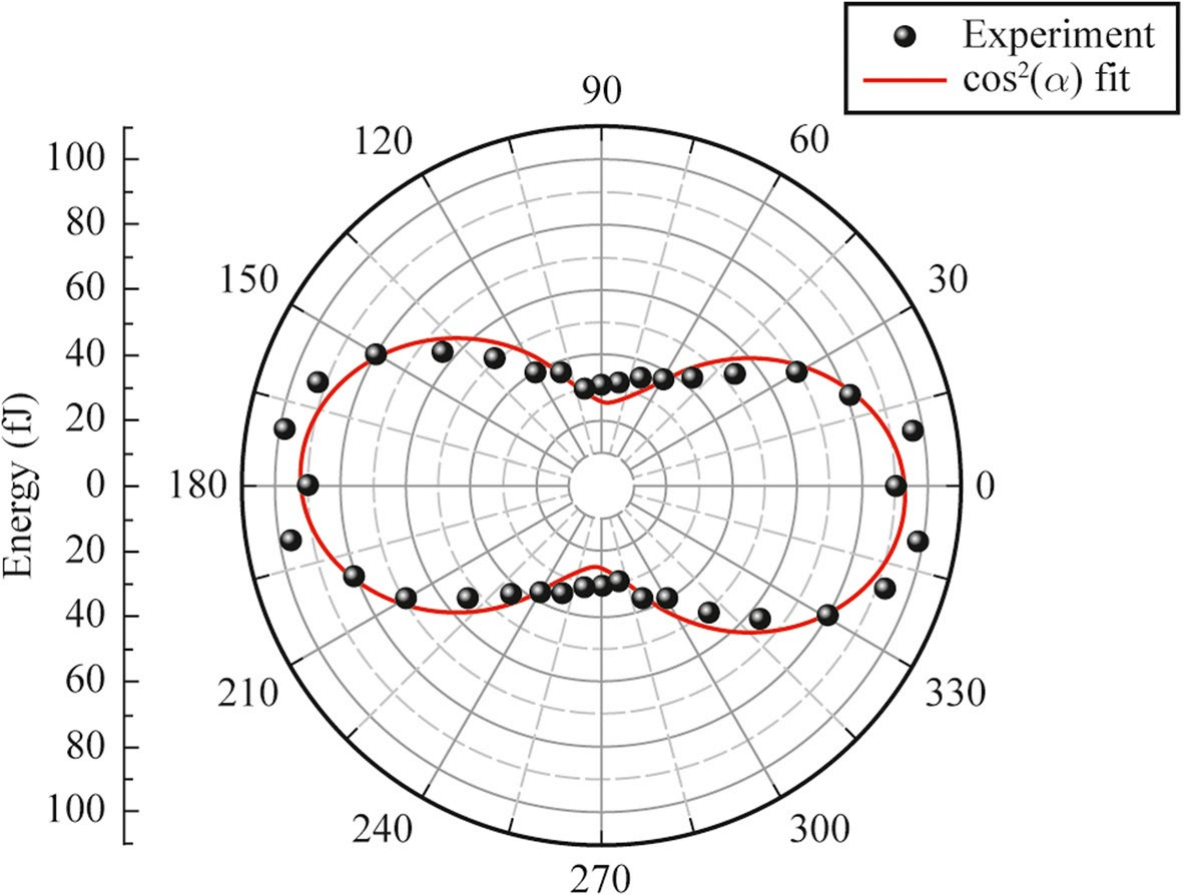
Energy of THz pulses as a function of wire grid polarizer orientation angle (dotted) and fit (red curve) by cosine function. Zero position corresponds to horizontal polarization transmission of polarizer

On the next step, we have determined the polarization state of the THz emission from the vacuum photodiode. The dependence of THz pulses energy as a function of a wire grid polarizer orientation angle *α* is presented below (see Fig. 6). One can see that the polarization of THz pulses is linear and horizontal. This phenomenon is in good agreement with numerical simulations. Moreover, the polarization state of THz pulses is not dependent on the polarization state of the optical excitation: first of all, it is governed by the direction of the current density in the photodiode. Some deviations between observed data and Malus law in Fig. 6 may be interpreted by following. In our model two ideally parallel electrodes (cathode and anode) with transversally applied electric field are considered. This configuration should provide generation of linearly polarized THz beams. In experimental realization there are several reasons for nonideal condition. First, atmospheric pressure bends the anode window. According to our estimations, displacement of central part of anode in comparison with the one near to vacuum flange is around several tens of microns, which is significantly lesser than the gap between anode and cathode, however, it may cause field lines to bend. Second, the cathode surface roughness also may alter electron trajectories and consequently THz pulses polarization. Moreover, we use wire grid polarizer for determination of THz beam polarization state. Since the period of wires is 100 µm, this polarizer is excellent for long wave component, however for THz waves shorter than its period (frequencies higher than 3 THz), this element does not operate as an polarizer. We have checked the extinction ration of the polarizer by introducing two polarizers in the THz beam. To this end first polarizer was oriented for maximal transmittance of THz pulses from photodiode and fixed at this orientation. Thereafter the same second polarizer was introduced after the first one, and measurements of THz pulse energy for collinear and crossed polarizer states have been done. Extinction ratio is around 50:1.

**Fig. 7:**
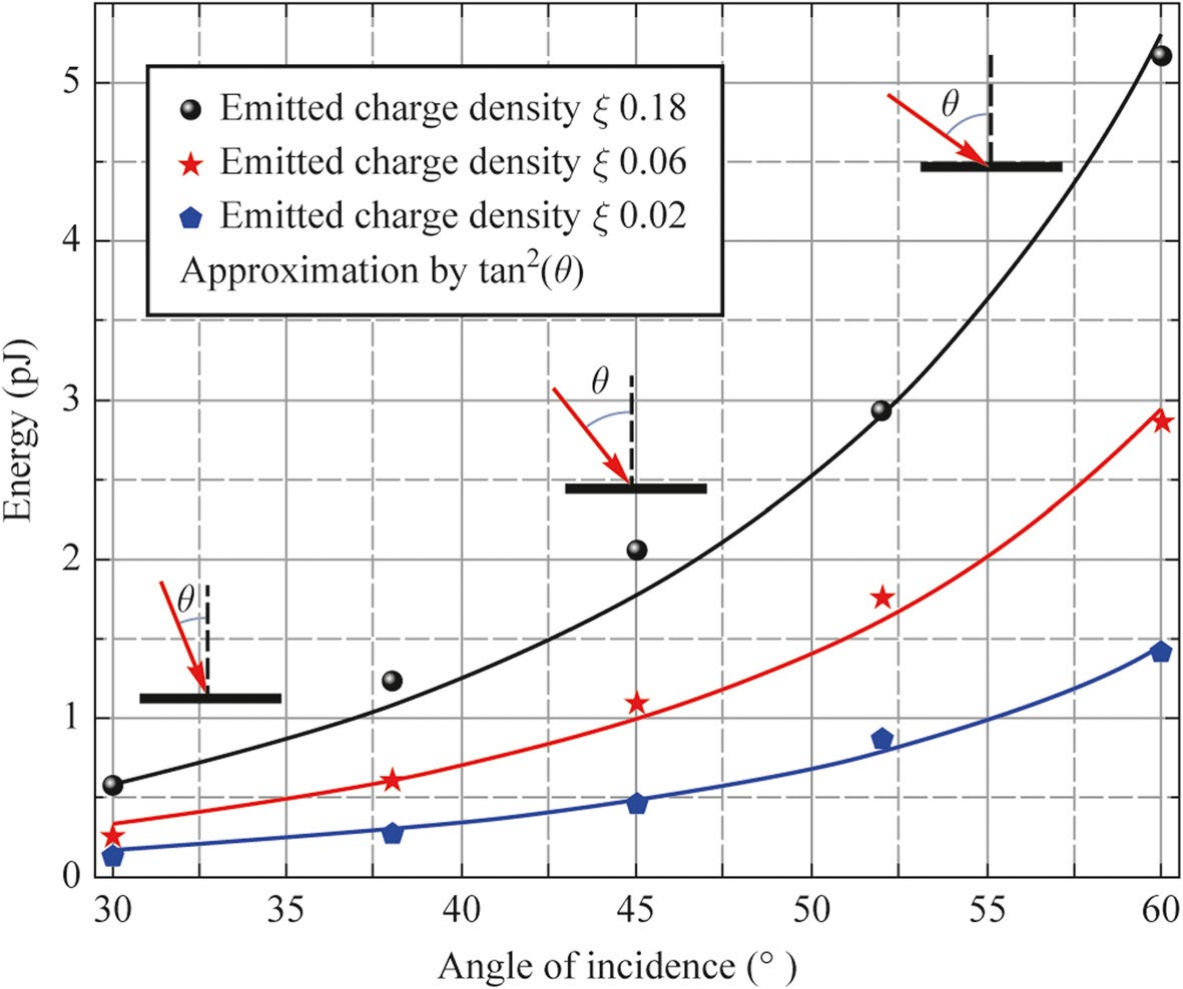
Dependence of THz pulse energy on incidence angle of optical radiation *θ* (dots) and approximation of experimental data by means of tangent function (solid lines)

We also have performed experiments directed on the dependence of the THz yield vs. the incidence angle *θ* of the optical excitation. In these measurements the photodiode has been oriented at different angles to the incident optical radiation. At the same angle on the reflection direction the PTFE lens and the detector have been set. The emitted charge value was the same for each series of angular measurements by the variation of the optical radiation energy. External electric field was set at the value of 45 kV/cm. According to Eqs. (1) and (2) the magnetic field strength is proportional to tan*θ*, hence one can expect that the measurements of THz pulses energy in terms of incident angle *θ* should have a tangent squared behavior. The results of the experiments (see Fig. 7) indeed demonstrate tangent squared dependence. However, it should be noted that the incidence angles more than 60° lead to clipping of the optical radiation on the photodiode aperture used in experiments. So, there could not be an infinite THz yield at the *θ* → 90° due to photodiode area limitation.

Our model does not consider any limit in the emitted charge density in terms of the incident optical radiation and its saturation. Indeed, a rise of incident angle up to 90° will increase an emitted surface area on the one hand and decrease the value of incident photon quantity density on the other hand. In this case an efficiency of source will decrease at the cos^2^ *θ* function. Nevertheless, the maximum THz yield in these experiments has been reached around 5.5 pJ.

**Fig. 8:**
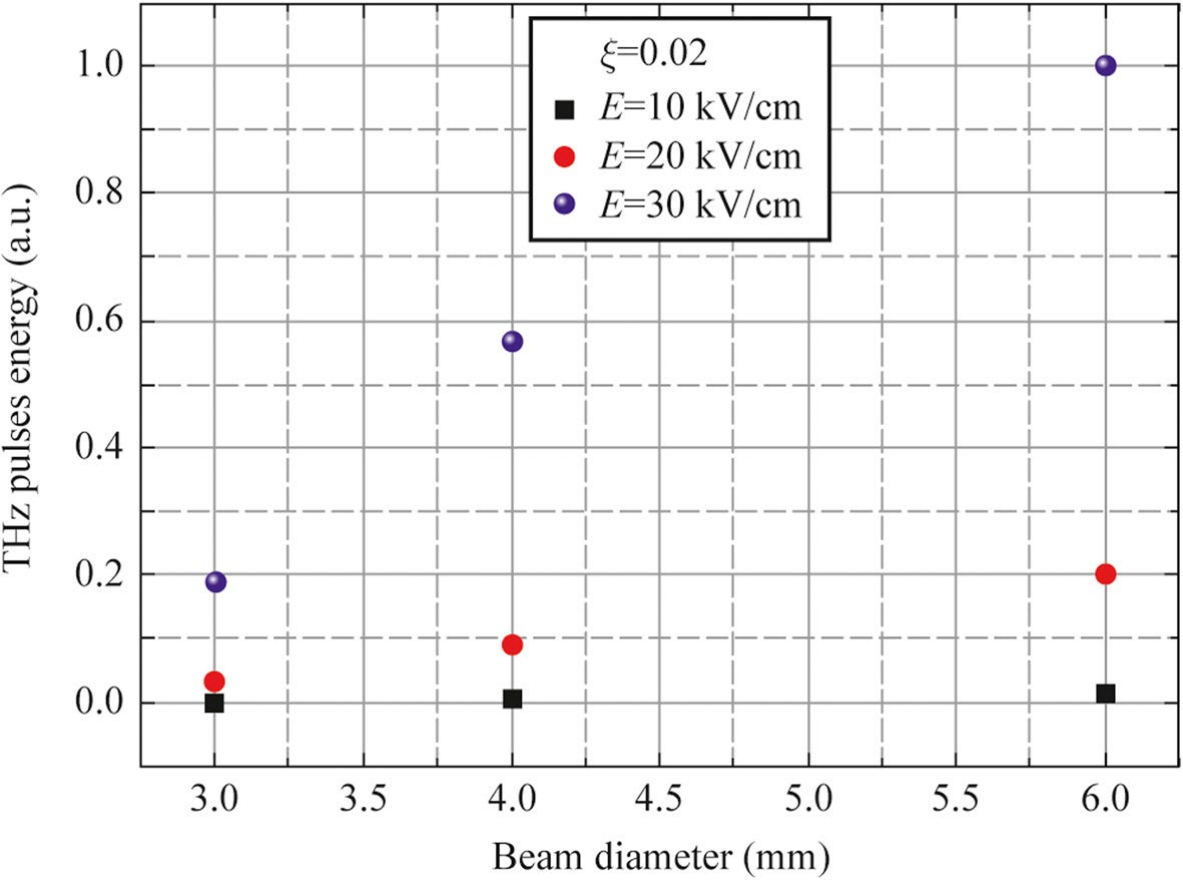
THz pulses energy as a function of laser beam diameter

We have also measured the dependence of THz pulses energy as a function of laser beam diameter (see Fig. 8).

To this end the initial laser beam has been expanded in two times by telescope for a more homogeneous excitation of photocathode. Thereafter an iris diaphragm, incerted into laser beam, has been used for control of beam diameter. This experiment has been performed at optical excitation 45° incident angle. Observed dependence demonstrates an increase in the output THz pulses energy vs. increase in laser spot diameter that agrees with the model in Ref. [18].

**Fig. 9:**
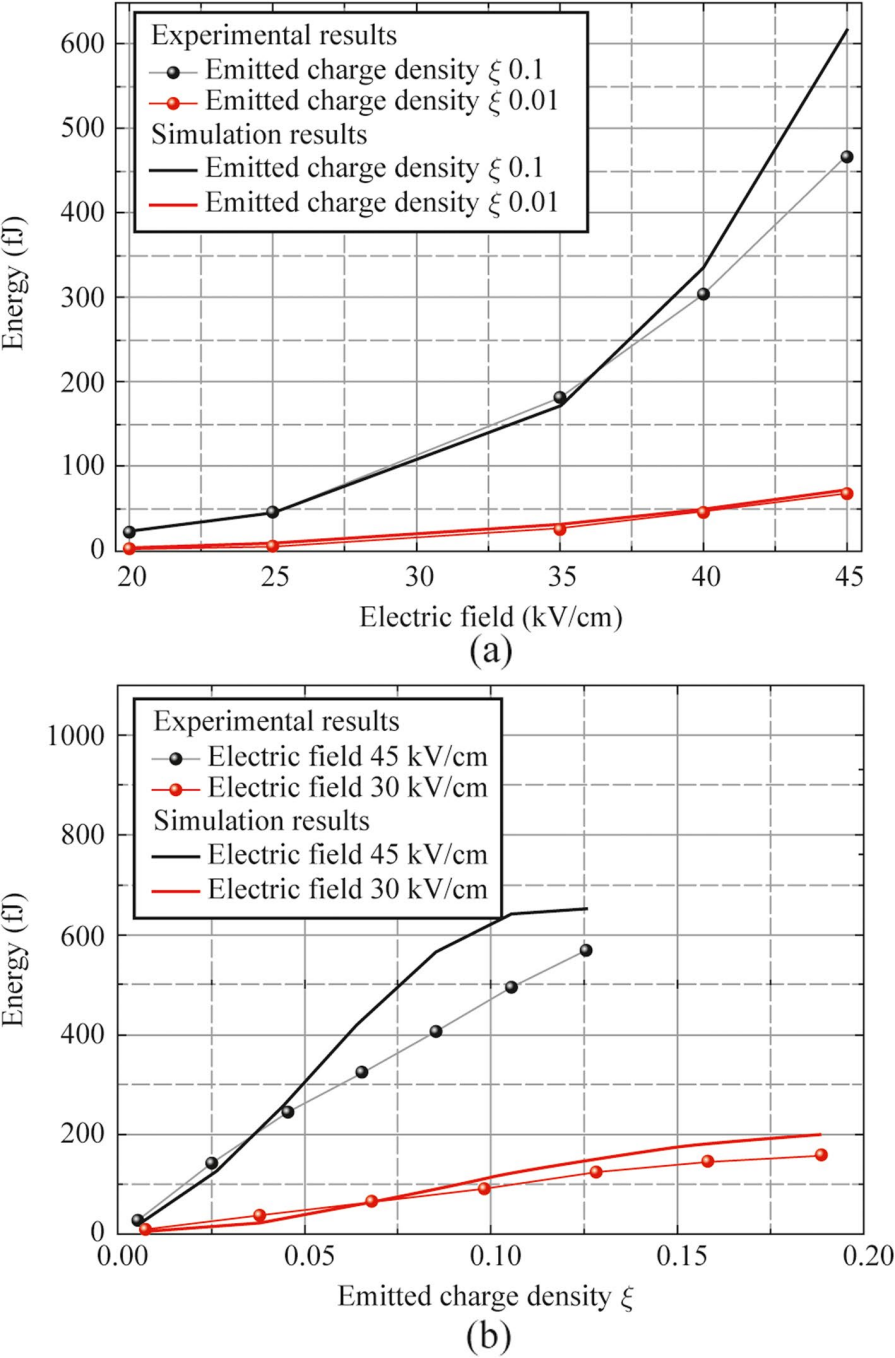
THz pulses energy as a function of applied **a** external electric field and **b** emitted charge density

The most important parameters for the vacuum photodiode – generator of pulsed THz radiation are the applied external electric field *E* = *U*/*d* and the emitted charge density *ξ*. The dependencies of THz pulse energies vs. external electric field and emitted charge density have been obtained for 45° incident angle of the optical radiation (see Fig. 9). For a comparison corresponding numerical simulations results are also introduced in Fig. 9.

In numerical simulations, EMR magnetic field waveforms have been calculated. Thereafter the power spectrum has been extracted. Finally, an integration of power components in THz spectral region corresponding to the detector operation range with transmission of the anode and the attenuators have been performed. Thus, the THz pulse energy has been obtained. One can see, that increase of *E* and *ξ* leads to more energetic THz pulses. Also, the measurements of THz pulses vs. emitted charge density do not demonstrate a square of electron charge dynamics, which may be concerned with nonuniform spectral detection of bolometer, limitation of spectral detection region of the detector (which is also included in simulations) and aperture Abbe limit. Statically breakdown by electric field makes some experimental limitation on applied electric fields. The 1 mm distance gap between cathode and anode was evacuate (typical pressure is around 10^−6^ mbar). This condition should increase a breakdown threshold in comparison with atmospheric pressure. The maximal presented experimental results correspond to an applied electric field of 45 kV/cm. Higher applied static fields lead to breakdown, which has been detected by significant increase of the electric current in cathode–anode electric circuit. However, the main limitation of the EMR generator in our experiments was a low quantum efficiency of the used photocathode. An increase of electron/photon ratio for photocathode should lead to a more effective generation of THz pulses.

## Conclusion

In this paper, we numerically and experimentally showed the possibility of the THz pulses generation in spectral range 0.1–3 THz under electron emission in the vacuum photodiode, consisted of a metallic cathode, illuminated by femtosecond laser pulses, and an anode opaque for the emitted electrons. We determine that the polarization state of generated pulses is determined by the direction of the current density. The increase of the incident angle of the optical radiation lead to a tangent squared rise of the THz pulses energy. Applying of higher external electric field and increase of the emitted charge density also provides more energetic THz pulses. All of presented results may be useful for the developing of new sources of THz radiation for tasks of broadband noninvasive tomography, imaging, radar and power effects on electronics.

## Data Availability

The data that support the findings of this study are available from the corresponding author, upon reasonable request.

## References

[1] Zhang XC, Xu J (2010). Introduction to THz Wave Photonics.

[2] Grischkowsky D, Keiding S, Van Exter M, Fattinger C (1990). Far-infrared time-domain spectroscopy with terahertz beams of dielectrics and semiconductors. J. Opt. Soc. Am. B.

[3] Chan WL, Deibel J, Mittleman DM (2007). Imaging with terahertz radiation. Rep. Prog. Phys..

[4] Jen CY, Richter C (2014). Sample thickness measurement with THz-TDS: resolution and implications. Int. J. Infrared Millim. Terahertz Waves.

[5] Ushakov, A., Chizhov, P., Bukin, V., Savel’ev, A., Garnov, S.: Broadband in-line terahertz 2D imaging: comparative study with time-of-flight, cross-correlation, and Fourier transform data processing. J. Opt. Soc. Am. B 35(5), 1159–1164(2018)

[6] Fattinger C, Grischkowsky D (1989). Terahertz beams. Appl. Phys. Lett..

[7] Obraztsov PA, Bulgakova VV, Chizhov PA, Ushakov AA, Gets DS, Makarov SV, Bukin VV (2021). Hybrid perovskite terahertz photoconductive antenna. Nanomaterials (Basel).

[8] Hamster, H., Sullivan, A., Gordon, S., Falcone, R.W.: Short-pulse terahertz radiation from high-intensity-laser-produced plasmas. Phys. Rev. E Stat. Phys. Plasmas Fluids Relat. Interdiscip. Topics 49(1), 671–677(1994) 10.1103/physreve.49.671 9961261

[9] Cook DJ, Hochstrasser RM (2000). Intense terahertz pulses by four-wave rectification in air. Opt. Lett..

[10] Ushakov, A.A., Chizhov, P.A., Andreeva, V.A., Panov, N.A., Shipilo, D.E., Matoba, M., Nemoto, N., Kanda, N., Konishi, K., Bukin, V.V., Kuwata-Gonokami, M., Kosareva, O.G., Garnov, S.V., Savel’ev, A.B.: Ring and unimodal angular-frequency distribution of THz emission from two-color femtosecond plasma spark. Opt. Express 26(14), 18202–18213(2018) 10.1364/OE.26.018202 30114100

[11] Ushakov, A.A., Panov, N.A., Chizhov, P.A., Shipilo, D.E., Bukin, V.V., Savel’ev, A.B., Garnov, S.V., Kosareva, O.G.: Waveform, spectrum, and energy of backward terahertz emission from two-color femtosecond laser induced microplasma. Appl. Phys. Lett. 114(8), 081102(2019)

[12] Mokrousova, D.V., Savinov, S.A., Seleznev, L.V., Rizaev, G.E., Koribut, A.V., Mityagin, Yu.A., Ionin, A.A., Nikolaeva, I.A., Shipilo, D.E., Panov, N.A., Ushakov, A.A., Savel’ev, A.B., Kosareva, O.G., Shkurinov, A.P.: Tracing air-breakdown plasma characteristics from single-color filament terahertz spectra. Int. J. Infrared Millim. Terahertz Waves 41(9), 1105–1113(2020)

[13] Chizhov, P.A., Volkov, R.V., Bukin, V.V., Ushakov, A.A., Garnov, S.V., Savel’ev-Trofimov, A.B.: Generation of terahertz radiation by focusing femtosecond bichromatic laser pulses in a gas or plasma. Quantum Electron. 43(4), 347–349(2013)

[14] Tcypkin, A.N., Ponomareva, E.A., Putilin, S.E., Smirnov, S.V., Shtumpf, S.A., Melnik, M.V., e, Y., Kozlov, S.A., Zhang, X.C.: Flat liquid jet as a highly efficient source of terahertz radiation. Opt. Express 27(11), 15485–15494(2019) 10.1364/OE.27.015485 31163744

[15] Hebling, J., Yeh, K.L., Hoffmann, M.C., Bartal, B., Nelson, K.A.: Generation of high-power terahertz pulses by tilted-pulse-front excitation and their application possibilities. J. Opt. Soc. Am. B 25(7), B6(2008)

[16] Vicario, C., Ovchinnikov, A.V., Ashitkov, S.I., Agranat, M.B., Fortov, V.E., Hauri, C.P.: Generation of 0.9-mJ THz pulses in DSTMS pumped by a Cr:Mg_2_SiO_4_ laser. Opt. Lett. 39(23), 6632–6635(2014) 10.1364/OL.39.006632 25490639

[17] Chai, X., Ropagnol, X., Ovchinnikov, A., Chefonov, O., Ushakov, A., Garcia-Rosas, C.M., Isgandarov, E., Agranat, M., Ozaki, T., Savel’ev, A.: Observation of crossover from intraband to interband nonlinear terahertz optics. Opt. Lett. 43(21), 5463–5466(2018) 10.1364/OL.43.005463 30383033

[18] Carron NJ, Longmire CL (1976). Electromagnetic pulse produced by obliquely incident X Rays. IEEE Trans. Nucl. Sci..

[19] Lazarev YN, Petrov PV (1995). Generation of an intense directed ultrashort electromagnetic pulse. JETP Lett..

[20] Lazarev YN, Petrov PV (1999). Microwave generation using a superluminal source. J. Exp. Theor. Phys..

[21] Klatt G, Hilser F, Qiao W, Beck M, Gebs R, Bartels A, Huska K, Lemmer U, Bastian G, Johnston MB, Fischer M, Faist J, Dekorsy T (2010). Terahertz emission from lateral photo-Dember currents. Opt. Express.

[22] Kononenko, V.V., Bukin, V.V., Komlenok, M.S., Zavedeev, E.V., Kononenko, T.V., Dezhkina, M.A., Ratnikov, P.P., Dolmatov, T.V., Chizhov, P.A., Ushakov, A.A., Konov, V.I., Garnov, S.V.: A diamond terahertz large aperture photoconductive antenna biased by a longitudinal field. Photonics 10(10), 1169(2023)

[23] Kondrat’ev, A.A.: Angular and spectral distributions of the broadband radiation of a photoemission source. Tech. Phys. 63(3), 434–437(2018)

[24] Lazarev YN, Petrov PV, Syrtsova YG (2004). Photoemission pulsed source of wide-band directional electromagnetic radiation. Tech. Phys..

[25] Potapov, A.V., Zavolokov, E.V., Kondrat’ev, A.A., Pkhaiko, N.A., Sorokin, I.A., Goncharenko, B.G., Luzanov, V.A., Salov, V.D.: Generation of ultrawideband electromagnetic radiation by a vacuum photodiode with anode with a sapphire input window. Tech. Phys. 66(3), 491–495(2021)

[26] Andreeva, V.A., Kosareva, O.G., Panov, N.A., Shipilo, D.E., Solyankin, P.M., Esaulkov, M.N., González de Alaiza Martínez, P., Shkurinov, A.P., Makarov, V.A., Bergé, L., Chin, S.L.: Ultrabroad terahertz spectrum generation from an air-based filament plasma. Phys. Rev. Lett. 116(6), 063902(2016) 10.1103/PhysRevLett.116.063902 26918992

